# Atomistic Design of Favored Compositions for Synthesizing the Al-Ni-Y Metallic Glasses

**DOI:** 10.1038/srep16218

**Published:** 2015-11-23

**Authors:** Q. Wang, J. H. Li, J. B. Liu, B. X. Liu

**Affiliations:** 1 Advanced Materials Laboratory, School of Materials Science and Engineering Tsinghua University, Beijing 100084, CHINA

## Abstract

For a ternary alloy system promising for obtaining the so-called bulk metallic glasses (BMGs), the first priority issue is to predict the favored compositions, which could then serve as guidance for the appropriate alloy design. Taking the Al-Ni-Y system as an example, here we show an atomistic approach, which is developed based on a recently constructed and proven realistic interatomic potential of the system. Applying the Al-Ni-Y potential, series simulations not only clarify the glass formation mechanism, but also predict in the composition triangle, a hexagonal region, in which a disordered state, i.e., the glassy phase, is favored energetically. The predicted region is defined as glass formation region (GFR) for the ternary alloy system. Moreover, the approach is able to calculate an amorphization driving force (ADF) for each possible glassy alloy located within the GFR. The calculations predict an optimized sub-region nearby a stoichiometry of Al_80_Ni_5_Y_15_, implying that the Al-Ni-Y metallic glasses designed in the sub-region could be the most stable. Interestingly, the atomistic predictions are supported by experimental results observed in the Al-Ni-Y system. In addition, structural origin underlying the stability of the Al-Ni-Y metallic glasses is also discussed in terms of a hybrid packing mode in the medium-range scale.

Bulk metallic glasses (BMGs) have attracted considerable interest due to their fundamental scientific significance as well as great potential for engineering applications^1^. To best utilize this class of materials, naturally, in the field of BMGs, the first priority issue is to clarify the formation mechanism, which could serve as guidance in synthesizing the desired glassy alloys^2^,^3^,^4^,^5^. In this respect, a terminology named glass-forming ability (abbreviated as GFA) has long been used in describing the glass formation. The definition of GFA, according to Schröers’ recent review^6^, could be expressed by “one can speak of a material’s glass-forming ability as being either inversely proportion to the critical cooling rate or proportional to its critical casting thickness”. In practice, researchers often prefer to use the critical casting thickness as a measure, as copper-mold casting has become a commonly used producing technique and the cooling rate could then be considered to be fixed. It follows that the larger the critical casting thickness, the larger the GFA of the obtained glassy alloy. Note that the GFA was defined only for a specific glassy alloy and by some technical parameters. From a scientific point of view, it is also of vital importance to give consideration to an alloy system, e.g., focusing on the ternary system in the present work, and in this regard, the issue is to predict a favored glass formation region, and even pinpoint an optimized sub-region, in its composition triangle, as such prediction would directly be a quantitative guidance for the materials design in synthesizing the desired metallic glasses.

Previously, several empirical/semi-empirical rules or criteria have been proposed and served as guidelines for the design of favored compositions for glass formation, of which the frequently cited are deep eutectic rule, size difference rule, Miedema’s model and so on^7^,^8^,^9^,^10^,^11^. When comparing with the experimental results, however, their predictions have often shown some limitations. To the present authors’ view, the limitations of the proposed criterion/rule are from their starting bases, e.g., the deep eutectic rule was based on the equilibrium phase diagram and the size difference rule was based on the atomic sizes of the constituent elements, and these starting bases have some restrictions in well reflecting the internal characteristics of the alloy system concerned^3^. The key is therefore to seek for a valid starting base and further develop an approach capable of clarifying the metallic glass formation in the specific alloy system. From a physical viewpoint, the interatomic potential of an alloy system is able to reasonably describe the major interactions involved in the system. Therefore, if a realistic interatomic potential is constructed and known, most of the physical and chemical properties of the system, including those related to the BMGs, can be deduced through relevant computations and simulations^3^,^12^.

In the family of BMGs developed so far, the Al-based BMGs constitute a significant member, as they show unique properties in many aspects, such as high specific strength and even good ductility^13^. Among Al-based BMGs, Al-TM-RE (TM = Ni, Cu, Fe, Co, etc.; RE = Y, Ce, Gd, La, etc.) systems are found to be the most promising^14^. However, experimental observations of glass formation in the Al-TM-RE systems are often found to be in conflict with currently available rules or criteria and an in-depth understanding is demanded^13^,^15^. In the present study, the Al-Ni-Y ternary alloy system is selected as a representative of the Al-TM-RE systems for developing the atomistic approach. We propose to take a newly constructed Al-Ni-Y interatomic potential as the starting base together with a relevant simulation route to develop an atomistic approach which is capable of not only clarifying the formation mechanism, but also predicting a favored glass formation region (abbreviated as GFR hereafter) in its composition triangle and an amorphization driving force (abbreviated as ADF hereafter) for each possible glassy alloy located inside the predicted GFR. The predicted GFR indicates the energetically favored alloy compositions, which could serve as guidance for the composition design in synthesizing BMGs. The predicted ADF, related to the energy difference between the glassy phase and the crystalline solid solution counterpart, could give hint to the readiness of metallic glass formation for a specific glassy alloy located in the GFR, and may somehow be correlated with the technical defined GFA by either obtainable size or applied cooling speed^6^. It should be noted that both the predicted GFR and ADF are derived from the Al-Ni-Y potential of the system, reflecting mainly in an energetic or thermodynamic aspect. In practice, using different glass producing techniques, one could obtain different experimentally identified glass formation regions. The technical defined GFA could also be influenced by dynamic factors, such as the viscosity of the liquid melt, atomic diffusivity in the liquid, overheating during producing, etc. Consequently, the practically observed glass formation regions and the so-called GFA could exhibit some fluctuation from the predicted/revealed characteristics.

In addition, the properties of the condensed matters are believed to correlate with their atomic structures^16^,^17^. For the Al-TM-RE systems, it is intriguing that adding a few atomic percent of TM or RE as a third element could dramatically affect the glass formation and properties of the base binary alloy systems^13^,^14^,^15^. This sensitive alloying effect has been explained by some thermodynamic or kinetic arguments, including the equilibrium phase diagram, Gibbs free energy and fragility^18^. These arguments, however, fall within the macroscopic domain, and a microscopic picture at an atomic-scale is needed^19^. Consequently, resolving the atomic structure, monitoring the delicate modification of the characteristic short- and medium-range orders with TM or RE addition, could improve the understanding of such a microscopic picture and help elucidate its underlying structural origin. Although several atomic structural models have been proposed in the past decades^20^,^21^,^22^,^23^, owing to the complexity and diversity of the internal interactions in various alloy systems^3^, the atomic packing details in metallic glasses are still matters of intense debate. Note that the knowledge concerning the atomic structure of the metallic glasses is not only of scientific interest, but also, if well-clarified, would lead to an improved understanding and in turn help in controlling the properties of the metallic glasses^16^,^24^. Consequently, besides the discussion of the prediction of GFR and ADF from the Al-Ni-Y potential, we will also discuss how to apply the interatomic potential to characterize the atomic structure of the Al-Ni-Y metallic glasses via relevant computations and simulations^3^,^12^,^15^.

## Results

### Construction of the Al-Ni-Y interatomic potential

To develop an atomistic approach, the construction of a realistic interatomic potential of an alloy system is of critical importance^25^. To the best of the authors’ knowledge, no interatomic potential has been published for the Al-Ni-Y system. In the present study, a set of the Al-Ni-Y interatomic potentials are constructed under a formulism proposed recently by the authors’ group, i.e., the smoothed and long-range second-moment-approximation of tight-binding (TB-SMA) scheme^26^.

By incorporating a binomial truncation function in the original TB-SMA potential^27^,^28^, the smoothed and long-range TB-SMA scheme solves the energy ‘jump’ problem when the atom pairs ‘cross’ the cutoff radius^29^ and eliminates the possible non-physical behaviors in the subsequent simulations. The smoothed and long-range TB-SMA scheme can be expressed as follows:


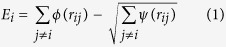



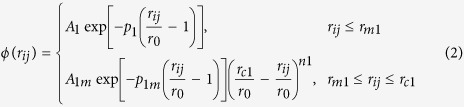



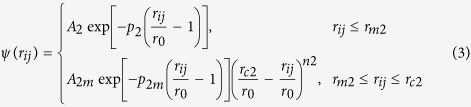


where 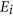 is the total potential energy of atom *i*, *ϕ* and *ψ* are called here the pair term and density term, respectively. *r*_m1_ and *r*_m2_ are the knots, and *r*_c1_ and *r*_c2_ are the cutoff radii of the pair term and density term, respectively. *n*_1_ and *n*_2_ are indices that should not be less than 3 and 5, respectively, to avoid discontinuity of the high derivatives. *A*_1_, *p*_1_, *A*_1m_, and *p*_1m_ and *A*_2_, *p*_2_, *A*_2m_, and *p*_2m_ are another eight adjustable potential parameters. The potential parameters are determined by fitting to the physical properties such as cohesive energies, lattice constants, elastic constants and bulk moduli of the elements as well as of the stable or virtual intermetallic compounds in each of the binary systems.

We now summarize the fitting results of the Al-Ni-Y potential. Table 1 displays the six sets of potential parameters for the Al-Ni-Y system. Table 2 gives the reproduced lattice constants, cohesion energies, elastic constants and bulk moduli for the stable structures of Al, Ni, and Y together with the corresponding experimental values^30^,^31^,^32^. It can be seen that the constructed potential can well reproduce the physical properties of the metals. To ensure that the potential could reflect the realistic atomic interactions involved in the systems, we have included a number of complicated compounds that are identified in the experiments (or found in the phase diagram)^33^,^34^,^35^,^36^,^37^,^38^,^39^, e.g., *oP*16-Al_3_Ni, *hP*5-Al_3_Ni_2_, *cF*24-Al_2_Y, *tP*20-Al_2_Y_3_, *oP*12-AlY_2_, *cF*24-Ni_2_Y, *oP*16-NiY_3_, and so on. Meanwhile, compounds with different compositions and crystallographic structures are also included, to reflect the atomic interactions in various chemical and structural environments. To aid the construction procedure, *ab initio* calculations are also conducted by the authors to derive the necessary physical properties of the compounds (detailed in Methods Section). Table 3 displays the reproduced lattice constants, cohesive energies, and bulk moduli of the stable or virtual compounds in the Al-Ni, Al-Y^15^, and Ni-Y systems, together with those obtained by the *ab initio* calculations as well as some available experimental lattice constants. From Table 3, one can see that the physical properties reproduced by the interatomic potential match well with those derived by *ab initio* calculations or experiments, confirming that the constructed Al-Ni-Y interatomic potential can well describe the energetic and structural characteristics of the alloy phases in the system.

**Figure 1:**
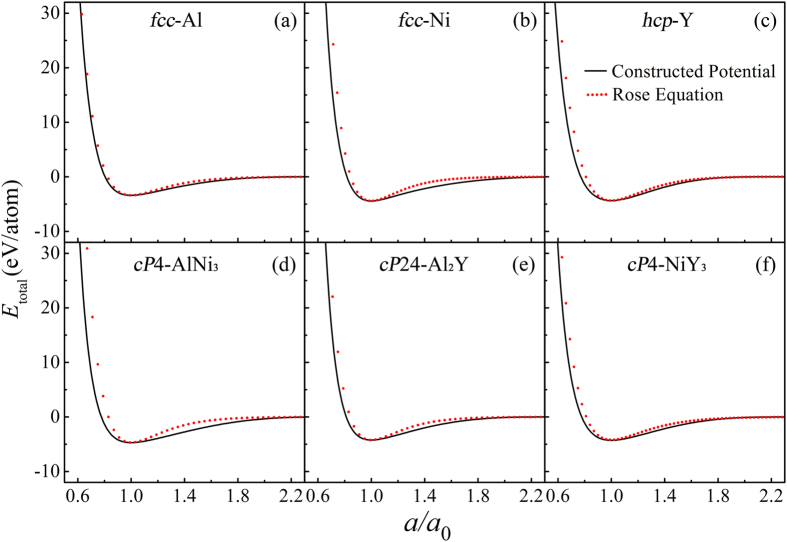
Equations of state (EOSs) calculated from the constructed potential (solid lines) and Rose equation (dotted lines) for (**a**) *fcc*-Al, (**b**) *fcc*-Ni, (**c**) *hcp*-Y, (**d**) *cP*4-AlNi_3_, (**e**) *cF*24-Al_2_Y, and (**f**) *cP*4-NiY_3_.

Another evaluation test for the relevance of the potential is to determine whether the potential can describe the atomic interactions at non-equilibrium conditions. A common practice is to derive the equation of state (EOS) from the potential and then compare it with the frequently used EOS in this field, i.e., the Rose equation, which is considered to be universal for all categories of solids^40^. Figure 1 shows the EOSs derived from the constructed Al-Ni-Y potential and the corresponding Rose equations for *fcc*-Al, *fcc*-Ni, *hcp*-Y, *cP*4-AlNi_3_, *cF*24-Al_2_Y, and *cP*4-NiY_3_, respectively^15^. Note that the EOSs have not been used in the fitting process and can therefore be considered as an external measurement for the relevance. From Fig. 1, one can see that the EOSs derived from the constructed potential agree well with the corresponding Rose equations, suggesting that the potential is relevant in describing the atomic interactions, even if the system is far from the equilibrium state. Meanwhile, the total energies are smooth in the whole range, without any ‘jumps’ or discontinuities, thus avoiding the appearance of non-physical behaviors in the simulations.

### Glass formation region of the Al-Ni-Y system

We now take the constructed Al-Ni-Y *n*-body potential as the starting base to develop an atomistic approach capable of clarifying the metallic glass formation.

To set up a relevant simulation route and model used in the simulations, here we first review the up-dated experimental results related to the metallic glass formation. Summarizing the experimental observations from various glass producing techniques, such as liquid melt quenching, ion beam mixing and solid-state amorphization, it is found that under these non-equilibrium glass producing techniques, the resultant alloy phase is either solid solution or glassy phase (i.e., in a disordered state), but not otherwise. This is because under these producing techniques, during which an effective cooling speed is estimated to be from 10^2^ to 10^13^ K/s, the available kinetic conditions are extremely limited and would certainly retard the ability of those complicated structured intermetallic compounds to nucleate and/or grow^3^. It follows that in the non-equilibrium producing process, the competing phase with the glassy phase is only the solid solution, which has one of the three simplest structures, i.e., *fcc*, *hcp*, or *bcc,* whereas the rather complicated intermetallic compounds, if exist, are excluded in this competition^3^,^41^,^42^. The above outcome from experimental observations suggests that predicting the GFR of a ternary alloy system can be converted into a scientific issue of splitting its composition triangle into two different types of regions, energetically favoring the glassy alloy and solid solution, respectively^3^. As shown above, a realistic Al-Ni-Y *n*-body potential is constructed and it governs the energetic states of all of the alloy phases, including the solid solution and glassy phase. It follows that the above scientific issue could be resolved by applying the constructed Al-Ni-Y potential to perform relevant atomistic simulations, in which solid solution models are used to compare the relative stability of the solid solution and its disordered counterpart over the entire composition triangle (detailed in Methods Section).

**Figure 2:**
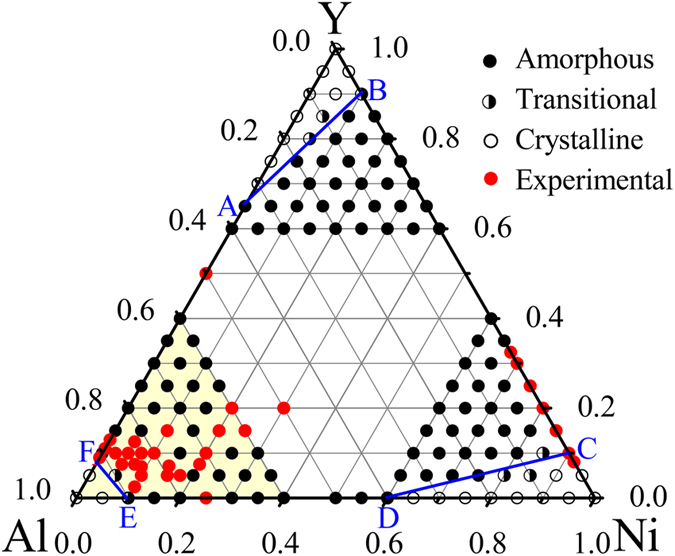
Glass formation region (ABCDEF) predicted from MC simulations at 300 K for the Al-Ni-Y ternary system. The light yellow shaded local area represents the Al-enriched alloy compositions, i.e., Al_*x*_Ni_*y*_Y_1−*x*−*y*_ (*x* = 60–100 at%, *y* = 0–40 at %) that have been widely studied.

Accordingly, we have set up the Al_*x*_Ni_*y*_Y_1−*x*−*y*_ solid solution models over the entire composition triangle. The constructed models are then allowed to evolve under Monte Carlo (MC) simulations^43^,^44^,^45^ within the isothermal-isobaric ensemble at 0 Pa and 300 K for 2 million steps, which is testified to be sufficient for the models to be fully relaxed (detailed in Methods Section). After sufficient simulation time, the initial solid solution models reach a relatively stable state, i.e., the drifts for all of the related dynamic variables are negligible. Inspecting the three-dimensional atomic configurations and pair correlation functions, it is revealed that when varying the compositions, the Al_*x*_Ni_*y*_Y_1−*x*−*y*_ solid solution models generally exhibit two different states, either preserving the initial crystalline state, or collapsing into a disordered state, corresponding to the formation of a glassy alloy. Simulations over the entire composition triangle allow locating the GFR of the Al-Ni-Y system, shown in Fig. 2. One can see that the composition triangle is split into two types of regions by three critical solubility lines AB, CD, and EF. When an alloy composition lies beyond AB, CD, and EF and moves towards one of three corners, the crystalline solid solution could remain stable. These regions are thus classified as the crystalline regions. When the composition falls in the central hexagonal region enclosed by ABCDEF, the solid solution becomes unstable and would spontaneously collapse into the disordered state. This lattice collapse, or say the crystalline-to-amorphous transition, is a result of the relaxation of the atomic-level stress when an adequate amount of solute atoms dissolve into the solvent lattice. Once the solute concentration exceeds a critical value, the severe lattice distortion could trigger a collective collapse of the crystalline lattice, turning into a more topologically stabilized disordered state than the crystalline state. This hexagonal region enclosed by ABCDEF is defined as the GFR of the Al-Ni-Y system. Within the GFR, the formation of the Al-Ni-Y metallic glasses is energetically favored. In addition, in the vicinity of the boundary of GFR, there are several models that reside in an ordered-disordered coexisting state. The formation of such coexisting state could be attributed to the stability of the solid solution and glassy phase being relatively close, leading the ordered and disordered states to coexist.

Figure 2 also indicates that for the Al-Ni and Al-Y binary sub-systems, the addition of a third element of Y or Ni helps the metallic glass formation. In the Al-Ni sub-system, the glassy phase could be obtained within a composition range of 10–60 at% Ni, and a few percent addition of Y would extend the GFR from one end to another in the composition triangle, suggesting that the ternary Al-Ni-Y metallic glasses could be formed at any combinations of Al and Ni. A similar case is also observed in the Al-Y sub-system, indicating that adding a third element of Ni also helps the metallic glass formation. Apparently, such a sensitive alloying effect obtained by adding a minor third element is of practical importance in synthesizing metallic glasses.

### Composition optimization for glass formation

We now further discuss the composition optimization for glass formation in the Al-Ni-Y system by calculating an amorphization driving force (ADF) for each glassy alloy inside the predicted GFR.

In practice, it has been observed that the Al-enriched metallic glasses with Al content over 60 at% feature good ductility^13^,^14^; we therefore focus on the Al-enriched corner, i.e., Al_*x*_Ni_*y*_Y_1−*x*−*y*_ (*x* = 60–100 at%, *y* = 0–40 at%) alloys for composition optimization. From an energetic point of view, the formation enthalpy difference between the glassy and solid solution phases (Δ*H*^glassy^ − Δ*H*^s.s.^) could serve as an amorphization driving force (ADF) for the specific alloy, whereas the formation enthalpy of the solid solution phase (Δ*H*^s.s.^) could act as a resistance against amorphization. We propose to define a parameter, 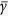, namely the normalized ADF for a specific glassy alloy inside the predicted GFR, and it can be expressed by


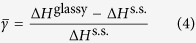


where Δ*H*^glassy^ and Δ*H*^s.s.^ are the formation enthalpies of the glassy and solid solution phases, respectively. Apparently, for an alloy, the parameter 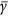 could be considered to be a measure of readiness in forming metallic glass. In the calculation of 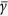, Δ*H*^glassy^ can be conveniently derived from MC simulations. Assuming that 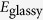 is the energy per atom of the Al_*x*_Ni_*y*_Y_1−*x*−*y*_ glassy phase and that 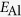, 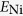, and 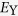 are the lattice energies of Al, Ni, and Y, respectively.


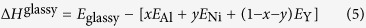


For Δ*H*^s.s.^, all of the solid solution models need to be relaxed to reach their respective minimum energies at various compositions. In relaxation, the MC box is set to be fixed, i.e., retaining the initial crystalline structures, whereas only atom displacement takes place in the process. The models are then relaxed for an adequate time period. Assuming that 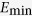
is the minimum energy per atom of the Al_*x*_Ni_*y*_Y_1−*x*−*y*_ solid solution, the formation enthalpy Δ*H*^s.s.^ of the solid solution could then be expressed by


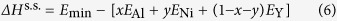


**Figure 3:**
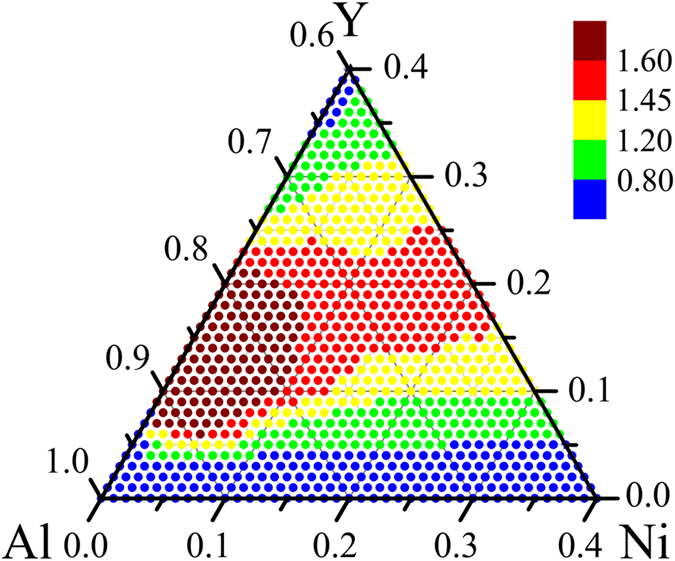
Contour map of the defined parameter γ, i.e., normalized amorphization driving force, calculated from MC simulations at 300 K for the AlxNiyY1-x-y (*x* = 60–100 at%, *y* = 0–40 at%) alloys, falling in the light yellow shaded Al-enriched area in Fig. 2.

The contour map of 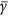 for the Al_*x*_Ni_*y*_Y_100−*x*−*y*_ (*x* = 60–100 at%, *y* = 0–40 at %) alloys is plotted in Fig. 3. Inspecting Fig. 3, it can be seen that when the compositions are located inside the area marked with red dots, their corresponding 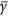 parameters are larger than those residing outside. From further calculations, a maximum value of 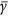 can be deduced at a stoichiometry of Al_80_Ni_5_Y_15_, and the surrounding sub-region marked with deep red dots could be considered as the optimized compositions for the Al-Ni-Y metallic glass formation. This means that from an energetic point of view, if an Al-Ni-Y alloy is designed with its composition located in this small sub-region, the obtained metallic glass would probably be prominently stable, and that from a kinetic point of view, this glassy alloy might be more easily obtained in practice than those located outside the sub-region.

## Discussion

### Comparison with the experimental observations

We now discuss the comparison of the predictions for the GFR and ADF by the atomistic approach with the experimental observations reported so far in the literature.

First, to validate the predicted GFR for the Al-Ni-Y alloy system, we have collected the available data^13^,^14^,^46^ and marked them in Fig. 2 with red dots. For example, Inoue *et al.* prepared Al-Ni-Y metallic glasses within the composition range of 3–22 at% Y and 4–33 at% Ni by melt-spinning^13^,^14^, and Yang *et al.* obtained an Al-enriched BMG around Al_86_Ni_8_Y_6_ by single roller melt-spinning^46^. One can see from Fig. 2 that the compositions of these experimentally obtained metallic glasses mostly fall within the predicted GFR, indicating that the proposed atomistic approach is quite reasonable in predicting/determining the GFR for the Al-Ni-Y system, allowing one to conveniently design appropriate compositions for synthesizing the Al-Ni-Y metallic glasses. Meanwhile, as illustrated by the yellow shaded area in Fig. 2, the compositions of the experimentally obtained metallic glasses mostly fall within the Al-enriched corner, suggesting that this area is indeed promising for searching for the optimized compositions for forming metallic glasses.

Second, it is of interest to determine if the atomistic prediction of the ADF for individual Al-Ni-Y alloys could somehow be correlated with the technically defined GFA, which is frequently cited in the literature. Inspecting Figs 2 and 3, one can see that experimentally measured compositions of the obtained Al-enriched metallic glasses are densely distributed in the red area in Fig. 3, within which the normalized ADF (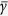) parameters are calculated to be larger than those compositions sitting outside. Furthermore, it is found that the Al contents of the best glass formers determined in the experiments are typically around 85–86 at%^46^,^47^,^48^,^49^. Interestingly, these experimental compositions mostly fall within the deep red sub-region (nearby the stoichiometry of Al_80_Ni_5_Y_15_), which is deduced to include the optimized compositions for the Al-Ni-Y metallic glass formation. It is noted that the pinpointed optimized compositions are defined by a small sub-region, but not a single number. This is because the above calculations/simulations are based on the constructed Al-Ni-Y *n*-body potential, in which a total of 6 × 15 parameters are fitted with the data from experiments or *ab initio* calculations, and the precision of the calculation/simulation results should be discussed with consideration of the inevitable errors involved. It is commonly accepted that in the atomistic simulations, the error is approximately 3–5%. A simple calculation then identifies a small composition sub-region around the maximum value of Al_80_Ni_5_Y_15_, and the compositions inside this small sub-region could be considered as the optimized compositions for the Al-Ni-Y metallic glass formation. From the above analyses, it can be seen that the experimental results are in support, in a qualitative or at least semi-quantitative manner, of the ADF predicted from the present atomistic approach for the Al-Ni-Y system. Moreover, as mentioned above, the predicted ADF represents the important energetic factor governing the metallic glass formation, yet it is not an exclusive one. For the technical defined GFA, it would be affected by a variety of factors in practice, and in addition to the energetic aspect, the liquid structure, viscosity, atomic diffusivity, as well as possible defects, impurities and microheterogeneities, etc., should all be considered. Therefore, the agreement in the present study between the atomistic prediction and technically measured parameters is quite reasonable. In other words, the predicted ADF provides a comparative measure, from an energetic point of view, for the GFA defined by some technical parameters, e.g., critical size or cooling speed.

In addition, it has been reported that the thermodynamic calculation method has also been employed to predict the glass formation in the Al-Ni-Y system^50^. However, it was found that the predictions exhibit considerable discrepancies with the experimental observations, especially in the Al-enriched corner. This might be attributed to the semi-empirical and phenomenological nature of the thermodynamic calculation method. The deviation between the thermodynamic predictions and experimental observations in the Al-enriched corner has also been revealed for other Al-TM-RE systems, such as the Al-Ni-(La,Ce) systems^50^. Moreover, a simple topologically-based prediction scheme has also been proposed to pinpoint the composition for Al-Ni-Y system and the predictions are quite acceptable^47^. However, this scheme is still based on a not so explicit starting base, i.e., cluster stability, and the predicted (local) optimal compositions are insensitive to the types of constituent atoms and similar for different alloy systems. Consequently, we have shown that the present atomistic approach, based on the interatomic potential, could be an improved approach in dealing with the issues of metallic glass formation in the ternary alloy systems.

Summarizing the above analyses, it is revealed that the atomistic prediction of the GFR and ADF in the Al-Ni-Y ternary alloy system is relevant and supported by the experimental observations. As the atomistic prediction is directly derived from the constructed Al-Ni-Y *n*-body potential, the relevant prediction could, in turn, provide additional evidence to the relevance of the constructed potential.

### Atomic Structure and stability of the metallic glasses

We then scrutinize the structural characteristics of the Al_80_Ni_5_Y_15_ metallic glass, which features the largest 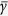, implying that it may have the largest phase stability as well.

**Figure 4:**
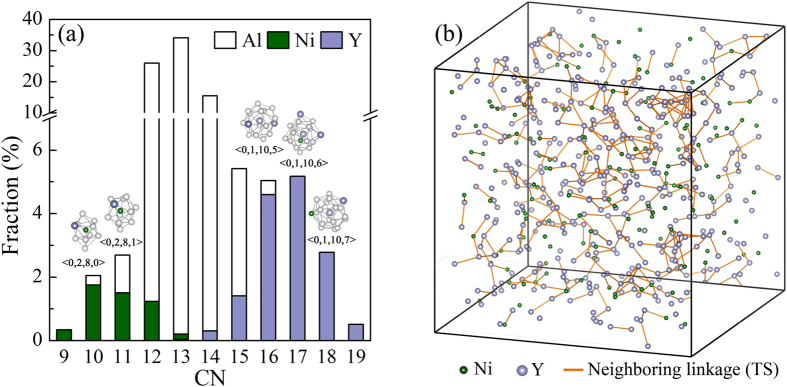
(**a**) Coordination number (CN) spectrum in the Al_80_Ni_5_Y_15_ metallic glass. It is observed that the CNs in Al_80_Ni_5_Y_15_ are well-distributed over a wide range, with three types of atoms covering different scopes of the whole landscape. The topologies of the dominant clusters centered with Ni and Y, respectively, with different sizes and Voronoi indices in Al_80_Ni_5_Y_15_ are also exhibited. (**b**) Snapshot of the distribution of the solute Ni- and Y-centered clusters in Al_80_Ni_5_Y_15_. Only central atoms of the clusters are plotted to obtain a clear presentation. The orange solid lines represent the neighboring linkages, i.e., tetrahedra-sharing (TS) linkages between the clusters, thereby ruling out the situation of ‘solute-solute avoidance’ as indicated by the typical quasi-equivalent packing model.

The short-range order (SRO) is characterized first. The Voronoi tessellation method is employed, and cell faces with smaller than 5% of the average face area are excluded to minimize the degeneracy problem and thermal vibration effects^45^. As presented in Fig. 4a, it can be seen that the CN spectra in Al_80_Ni_5_Y_15_ is distributed over a wide range, with three types of atoms covering different scopes of whole landscape, i.e., Ni dominates in CNs of 9–11 (average 9.8), Al dominates in 12–15 (average 13.3), and Y dominates in 16–19 (average 17.2). As the Al-Ni-Y system is a typical system comprised of small-, medium- and large-sized atoms, i.e., the Goldschmidt radii of Ni, Al and Y are 0.124 nm, 0.143 nm and 0.180 nm, respectively, thus, naturally, the larger radii of Y permit accommodate more atoms in the neighboring shells and lead to larger CNs, followed by Al, and then Ni. When forming metallic glasses, clusters of different sizes can better coordinate in space and efficiently fill the sites in the disordered structure, leading to the increase in packing density as well as the enhancement of phase stability. Furthermore, inspecting the distribution of Voronoi clusters, it is found that Ni and Y, as solutes, are mainly, but not strictly, surrounded by solvent Al as nearest-neighbors and mostly form Kasper-type clusters, such as Ni-centered <0, 2, 8, 0> and Y-centered <0, 1, 10, 6>. These solute-centered clusters can be considered as the building blocks in Al_80_Ni_5_Y_15_. However, this is comparable but slightly different from the scheme of solute-centered quasi-equivalent packing^21^, as no particular “solute-solute avoidance” is observed in Al_80_Ni_5_Y_15_. In previous studies, for the Al-Ni-La metallic glasses, such as Al_89_Ni_5_La_6_ with a larger Al content of 89 at%, the solute Ni and La are almost totally avoided, exhibiting a large extent of chemical SRO^51^. However, in the present study, Ni and Y atoms are more close to random packing, with a Warren-Cowley parameter ~0. The packing of Ni and Y is revealed in Fig. 4b, where the neighboring linkages are displayed. This behavior can be attributed to the fact that the mixing enthalpy of Ni-Y is also largely negative, i.e., −31 kJ/mol^11^, which is comparable to those of Al-Ni and Al-Y, i.e., −22 and −38 kJ/mol, respectively^11^. Therefore, Ni and Y are not necessarily neighbored exclusively by Al and Ni-Y neighboring is also stabilized energetically. Meanwhile, as the total content of Ni and Y are up to 20 at% in Al_80_Ni_5_Y_15_, which is larger than typical solute-lean metallic glasses^51^, a certain extent of neighboring between Ni-Y is also expected from the nominal composition.

**Figure 5:**
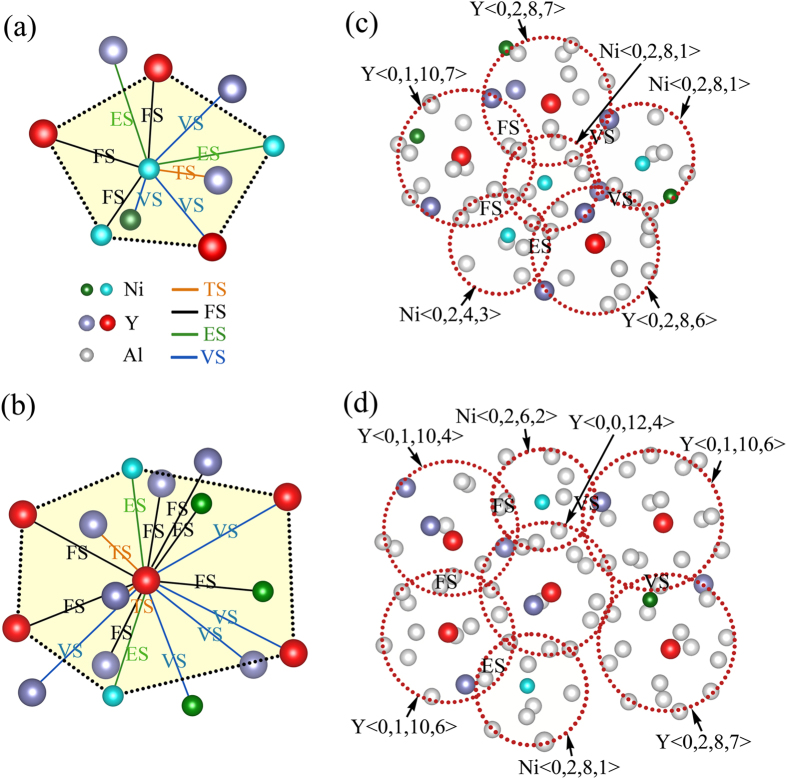
Hybridized packing mode of MRO in the Al_80_Ni_5_Y_15_ metallic glass. (**a,b**) Typical packing of the surrounding solute-centered clusters around the Ni and Y clusters, respectively, via vertex- (VS), edge- (ES), face- (FS) and tetrahedra-sharing (TS) linkages. Representative icosahedral-like (five-fold) and fcc-like (six-fold) arrangements are highlighted by black dotted lines. Only the central atoms in the clusters are plotted to achieve a clear presentation. (**c,d**) Close-up view of the highlighted icosahedral-like and fcc-like packing around the Ni and Y clusters, respectively.

Characterizing SRO alone, neglecting the heterogeneous correlations among various types of clusters that extend to the next level of hierarchy, i.e., medium-range and beyond, is insufficient to explain the structure-property relationship of metallic glasses^52^,^53^. A microscopic picture of medium-range order (MRO) can then be resolved by the percolated network formed by the solute-centered clusters. In MRO, the clusters adopt dense arrangements, by vertex- (VS), edge- (ES), face- (FS) and tetrahedra-sharing (TS) linkages^54^. In Fig. 5a,b, typical packing of neighboring clusters around Ni and Y clusters is illustrated, indicating that the VS, ES, FS and TS linkages are collectively hybridized in achieving the efficient packing in the metallic glass. In Al_80_Ni_5_Y_15_, the Ni-centered clusters typically have 10–13 solute-centered clusters as neighbors, whereas the Y-centered clusters often have 15–18 clusters around, suggesting more compact packing around Y clusters. Scrutinizing the packing of solute-centered clusters, a hybridized packing mode is observed, of which the icosahedral-like (five-fold) and fcc-like (six-fold) arrangements are most prevalent, as highlighted by the black dotted lines in Fig. 5a,b, and the higher-coordinated fcc-like arrangements are more populated around Y clusters. This observation agrees with our previous findings for the other Al-TM-RE systems^15^. The hybridized packing mode cannot be fully covered by previously proposed structural models, which often suggest a single packing mode for simplicity^16^,^20^,^21^. Our findings suggest that despite these structural models capturing the efficient packing nature of MRO, they are still providing an idealized, or over-simplified, picture. For real-world metallic glasses, due to the distinct atomic sizes and chemical interactions involved, MRO has its intrinsic complexity, just as revealed by the hybridized packing mode for the Al-Ni-Y metallic glasses. A close-up view of the highlighted icosahedral-like and fcc-like packing is further presented in Fig. 5c,d, respectively. This complexity of MRO creates more opportunities for space tiling, facilitates constituting a well-percolated network that can serve as the reinforced ‘backbone’, and eventually leads to the improved stability of the metallic glasses^49^.

To summarize, we have shown that by taking a proven realistic Al-Ni-Y *n*-body potential as the starting base to conduct series simulation under a relevant route over the entire composition triangle, an atomistic approach is developed. The atomistic approach clarifies that the physical origin of the metallic glass formation is the collapsing of solid solution when the solute concentration exceeds the critical value and predicts an energetically favored glass formation region (abbreviated as GFR) of the ternary alloy system. Moreover, the atomistic approach further predicts an amorphization driving force (abbreviated as ADF) for each alloy located within the GFR. The predicted ADF could be a comparative measure, from an energetic perspective, of the so-called glass-forming ability (known as GFA), which has long been used in the field, yet was defined by some technical parameters, e.g., critical size of the obtainable glass. In addition, structural analysis reveals that a hybridized packing mode exists in the Al-Ni-Y metallic glasses, manifesting the intrinsic complexity of MRO and relevantly interpreting the phase stability of the Al-Ni-Y metallic glasses. Obviously, the approach presented here could serve as an important guide in designing appropriate alloy compositions for synthesizing ternary BMGs and in finding new possible BMG formers as well.

## Methods

### Construction procedure of the Al-Ni-Y potential

In the present study, a set of the Al-Ni-Y *n*-body potentials is constructed under the smoothed and long-range TB-SMA formulism. Concerning the atomic interactions in the Al-Ni-Y system, there should be six sets of potentials, i.e., three sets for Al-Al, Ni-Ni and Y-Y, and three sets for Al-Ni, Al-Y and Ni-Y. The latter three sets are referred to as cross potentials, which describe interactions between dissimilar atoms. For each set of potential, there are 15 potential parameters to be fitted, i.e., <*p*_1_, *A*_1_, *r*_m1_, *n*_1_, *p*_1m_, *A*_1m_, *r*_c1_, *p*_2_, *A*_2_, *r*_m2_, *n*_2_, *p*_2m_, *A*_2m_, *r*_c2_, *r*_0_>. Specifically, the potential parameters of Al-Al, Ni-Ni and Y-Y are determined by fitting them to the experimental properties of the Al, Ni and Y metals at 0 K, such as the cohesive energy, lattice constants, elastic constants and bulk moduli^30^,^31^,^32^. The parameters of Al-Ni, Al-Y and Ni-Y cross potentials are determined by fitting them to the physical properties of several stable or virtual intermetallic compounds in each of the binary systems. However, there are always few available property data of the related compounds that could be used in the fitting procedure. To solve this problem, *ab initio* calculations are then conducted by the authors to derive the necessary physical properties of the compounds and assist the potential construction.

The *ab initio* calculations are carried out using the Cambridge serial total energy package (CASTEP)^55^,^56^ based on density functional theory (DFT). During the *ab initio* calculations, the exchange-correlation energy functional is described by the established Perdew-Wang (PW91) version of the generalized gradient approximation (GGA)^57^,^58^, and the ion-electron interactions are treated by the ultrasoft Vanderbilt pseudopotential scheme (US-PP)^59^. The cutoff energy is chosen to be 500.0eV, and the Brillouin zone is sampled using the Monkhorst-Pack method^60^ with nearly constant *k*-point densities for each calculation, roughly equivalent to a 12 × 12 × 12 mesh for a conventional *fcc* unit cell. These parameters are shown to be sufficient for convergence of the calculations for the structures in this work. Each structure is firstly optimized with respect to the external degree(s) of freedom as well as the internal degree(s) of freedom (if any) of the unit cell as permitted by the space-group symmetry of the crystalline structure. The lattice constants and total energies of the optimized structures are then obtained, and the elastic constants and bulk moduli can be derived by fitting the stress-strain relationship.

### Simulation model

To compare the relative stability of the solid solution and its amorphous counterpart, the solid solution models are commonly employed as the simulation models^3^. Two types of solid solution models, i.e., *fcc* and *hcp*, are established for the Al-Ni-Y system. For the *fcc* models, the [100], [010], and [001] crystalline directions are parallel to the *x*, *y*, and *z* axes, respectively. For the *hcp* models, the [100], [120], and [001] crystalline directions are parallel to the *x*, *y*, and *z* axes, respectively, and the *hcp* models can be considered to be built of equivalent orthorhombic cells. Each solid solution model consists of 2916 atoms, with periodic boundary conditions adopted in three Cartesian directions. To conduct a thorough investigation on the entire compositional phase-space, we have established Al_*x*_Ni_*y*_Y_1−*x*−*y*_ models over the entire composition triangle. The lattice parameters of the Al_*x*_Ni_*y*_Y_1−*x*−*y*_ solid solution models start out following the Vegard’s law. In setting up the solid solution models, solute atoms are added by random substitution of a certain number of solvent atoms to reach a desired concentration.

### Monte Carlo simulation

The established solid solution models are then allowed to evolve under Monte Carlo (MC) simulations within the isothermal-isobaric ensemble at 0 Pa and 300 K for 2 million steps, which is shown to be sufficient for the models to be fully relaxed. In order to minimize the possible random error, the MC simulations have been independently performed for five times and the calculation results are averaged. During the simulations, structural changes occurring in the models are monitored by the three-dimensional (3-D) atomic configuration, which can visually reflect the state of the system, and the pair correlation function, which is commonly recognized as firm evidence to identify the glassy phase.

The details of the present MC simulations are briefly described as follows. In MC simulations, there are two types of moves: atom displacement and box deformation. For atom displacement, the simulation system can be treated as a canonical ensemble at constant *NVT*, i.e., the shape and volume of the box are fixed. An atom *i* is chosen at random and given a uniform random displacement, 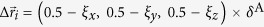, to a new trial position. Here, *ξ*_*x*_, *ξ*_*y*_ and *ξ*_*z*_ are three random numbers uniformly distributed in the range (0, 1]. *δ*^A^ is the amplitude of this trial move. The trial move is accepted with a probability given by 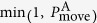. Here, 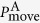 is the ratio of the probabilities of the new and old state


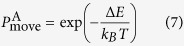


where Δ*E* = *E*^trail^ − *E* is the energy change resulting from the trial move of atom *i*. *k*_*B*_ and *T* are the Boltzmann constant and the temperature of the simulation system, respectively.

For box deformation, the fractional coordinates of atoms in the box are fixed. The state of the simulation box is defined by the matrix ***h***, from which the trial state ***h***^trail^ is generated by the following relations,


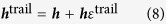



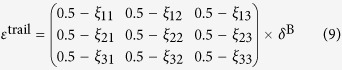


where *ε*^trail^ is the trial strain tensor applied to the box. *ξ*_*ij*_ are random numbers uniformly distributed in the range (0, 1]. *δ*^B^ is the amplitude of this trial move. The trial move is accepted with a probability given by 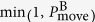. Here, 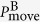 is the ratio of the probabilities of the new and old states


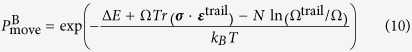


where Δ*E* = *E*^trail^ − *E* is the energy change that results from the trial move of the box. ***σ*** is the applied stress tensor. _Ω_^trail^ is the volume of the box in the trial state. When only hydrostatic pressure is applied, 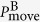 can be computed by


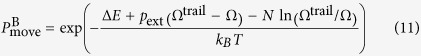


One can see from Eq. (9) that there are 6 independent elements in the strain tensor. To monitor the internal stress or pressure of the system, the 6 strains are separately applied to the box in a MC step and therefore the internal stress can be synchronously computed by


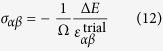


For a system consisting of *N* atoms, there are a total of *N* + 6 trial moves; *N* moves for atoms and 6 moves for the box. The *N* + 6 trial moves are randomly chosen to be carried out in an MC step. In addition, if a move is rejected, the old state is recounted. The amplitudes of trial moves *δ*^A^ and *δ*^B^ are also adjusted so that the acceptance ratios of the trial moves are kept around 50%.

## Additional Information

**How to cite this article**: Wang, Q. *et al.* Atomistic Design of Favored Compositions for Synthesizing the Al-Ni-Y Metallic Glasses. *Sci. Rep.* **5**, 16218; doi: 10.1038/srep16218 (2015).

## Figures and Tables

**Table 1:** Parameters of the constructed Al-Ni-Y interatomic potential under the proposed smoothed and long-range second-moment approximation of tight binding (TB-SMA) scheme.

	Al-Al	Ni-Ni	Y-Y	Al-Ni	Al-Y	Ni-Y
*p*_1_	8.776460	11.08757	7.293850	4.297973	11.36598	11.27927
*A*_1_	0.402184	0.287580	0.527193	1.011303	0.320283	0.154233
*r*_m1_	2.764394	1.976092	3.118844	1.582281	3.895313	2.725342
*n*_1_	4	4	4	4	4	4
*p*_1m_	2.558558	4.485833	2.254337	1.547865	1.415430	1.352604
*A*_1m_	2.917212	8.372519	2.761409	0.696275	1.741822	17.85251
*r*_c1_	4.607023	3.486092	6.014946	5.477747	5.204398	3.962593
*p*_2_	5.249466	3.669412	3.855623	3.665545	3.536090	3.513034
*A*_2_	4.738155	4.991288	8.470387	12.47185	6.520435	5.462924
*r*_m2_	3.786874	2.803510	2.847003	2.602315	2.906232	2.825342
*n*_2_	5	5	5	5	5	5
*p*_2m_	0.000477	0.000695	0.000578	0.000635	0.000481	0.000635
*A*_2m_	1.114067	0.671240	5.383636	2.927637	1.687934	1.236981
*r*_c2_	6.515324	6.200000	7.579420	6.256209	7.511561	7.196209
*r*_0_	2.864321	2.492155	3.648736	2.678238	3.256528	3.070445

*A*_1_ and *A*_1m_ are expressed in eV, *A*_2_ and *A*_2m_ are expressed in eV^2^, *r*_m1_, *r*_c1_, *r*_m2_, *r*_c2_ and *r*_0_ are expressed in Å.

**Table 2:** Lattice constants (*a* and *c*), cohesion energies (*E*_c_), elastic constants (*C*_ij_), and bulk moduli (*B*_0_) of Al, Ni, and Y fitted by the constructed potential and observed from experiments (Ref. 30, 31).Lattice constants *a* and *c* are expressed in Å, cohesion energies *E*_c_ in eV/atom, elastic constants *C*_ij_ and bulk moduli *B*_0_ in Mbar.

	*fcc*-Al		*fcc*-Ni		*hcp*-Y	
	Fitted	Expt.	Fitted	Expt.	Fitted	Expt.
*a*	4.051	4.050^a^	3.524	3.517^a^	3.650	3.648^a^
*c*					5.958	5.732^a^
*E*_c_	3.387	3.390^b^	4.423	4.440^b^	4.361	4.370^b^
*C*_11_	0.821	1.067^a^	2.385	2.418^a^	0.639	0.779^a^
*C*_12_	0.705	0.604^a^	1.630	1.550^a^	0.348	0.285^a^
*C*_13_					0.294	0.210^a^
*C*_33_					0.703	0.769^a^
*C*_44_	0.289	0.283^a^	1.217	1.242^a^	0.108	0.243^a^
*B*_0_	0.743	0.722^a^	1.882	1.860^a^	0.428	0.415^a^

^a^Ref. 30.

^b^Ref. 31.

**Table 3:** Lattice constants (*a*, *b*, and *c*), cohesive energies (*E*_c_), and bulk moduli (*B*_0_) of the Al-Ni, Al-Y and Ni-Y compounds reproduced from the constructed potential (first line) and derived from *ab initio* calculations (second line) together with some available experimental data for the lattice constants of the compounds (Ref. 33-39).

Compounds		Al_3_Ni	Al_3_Ni_2_	AlNi_3_	Al_2_Y	Al_2_Y_3_	AlY_2_	Ni_3_Y	Ni_2_Y	NiY_3_	NiY_3_
Space group		*Pnma*	*P* 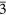 *m1*	*P* 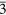 *mm*	*Fd* 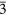 *m*	*P4* 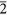 */mnm*	*Pnma*	*Pm* 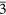 *m*	*Fd* 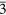 *m*	*Pm* 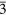 *m*	*Pnma*
*a* or *a*,*c* or *a*,*b*,*c*	Potential	6.732,7.556,4.927	4.145,5.060	3.618	8.080	8.451,7.778	6.879,5.254,9.663	3.903	7.250	3.456	7.308,9.427,6.375
	*Ab initio*	6.634,7.398,4.824	4.047,4.920	3.577	7.910	8.280,7.678	6.652,5.130,9.543	3.967	7.310	3.517	7.053,9.704,6.461
	Expt.	6.618,7.368,4.814^a^	4.039,4.903^b^		7.858^c^	8.239,7.648^d^	6.629,5.087,9.473^e^		7.181^f^		6.920,9.490,6.360^g^
*E*c	Potential	4.082	4.317	4.706	4.227	4.334	4.353	4.507	4.695	4.288	4.439
	*Ab initio*	4.087	4.460	4.660	4.242	4.331	4.349	4.498	4.788	4.203	4.581
*B*_0_	Potential	0.807	0.753	1.046	0.933	0.787	0.702	0.962	1.320	0.500	0.546
	*Ab initio*	1.058	1.072	1.329	0.856	0.717	0.614	1.228	1.215	0.490	0.788

Lattice constants *a*, *b* and *c* are expressed in Å, cohesive energies *E*_c_ in eV/atom, and bulk moduli *B*_0_ in Mbar.

^a^Ref. 33

^b^Ref. 34

^c^Ref. 35

^d^Ref. 36

^e^Ref. 37

^f^Ref. 38

^g^Ref. 39.

## References

[b1] Inoue A. & Takeuchi A. Recent development and application products of bulk glassy alloys. Acta Mater. 59, 2243–2267 (2011).

[b2] Li Y., Guo Q., Kalb J. A. & Thompson C. V. Matching Glass-Forming Ability with the Density of the Amorphous Phase. Science 322, 1816–1819 (2008). 19095935 10.1126/science.1163062

[b3] Li J. H., Dai Y., Cui Y. Y. & Liu B. X. Atomistic theory for predicting the binary metallic glass formation. Mater. Sci. Eng. R. 72, 1–28 (2011).

[b4] Kang D.-H. *et al.* Interfacial Free Energy Controlling Glass-Forming Ability of Cu-Zr Alloys. Sci. Rep. 4, 5167 (2014). 24893772 10.1038/srep05167 PMC4044622

[b5] Yu C. Y., Liu X. J., Lu J., Zheng G. P. & Liu C. T. First-principles prediction and experimental verification of glass-forming ability in Zr-Cu binary metallic glasses. Sci. Rep. 3, 2124 (2013). 23821016 10.1038/srep02124 PMC3699808

[b6] Schroers J. Bulk Metallic Glasses. Phys. Today 66, 32–37 (2013).

[b7] Turnbull D. Under what conditions can a glass be formed? Contemp. Phys. 10, 473–488 (1969).

[b8] Egami T. & Waseda Y. Atomic size effect on the formability of metallic glasses. J. Non-cryst. Solids. 64, 113–134 (1984).

[b9] Liu B. X., Johnson W. L., Nicolet M. A. & Lau S. S. Structural difference rule for amorphous alloy formation by ion mixing. Appl. Phys. Lett. 42, 45–47 (1983).

[b10] Johnson W. L. Bulk Glass-Forming Metallic Alloys: Science and Technology. Mrs. Bull. 24, 42–56 (1999).

[b11] De Boer F. R., Boom R., Mattens W., Miedema A. & Niessen A. Cohesion in Metals: Transition Metal Alloys. (Elsevier Science Publishers B. V, 1988).

[b12] Li J. H. *et al.* Interatomic potentials of the binary transition metal systems and some applications in materials physics. Phys. Rep. 455, 1–134 (2008).

[b13] Inoue A. Amorphous, nanoquasicrystalline and nanocrystalline alloys in Al-based systems. Prog. Mater. Sci. 43, 365–520 (1998).

[b14] Inoue A., Ohtera K., Tsai A.-P. & Masumoto T. New Amorphous Alloys with Good Ductility in Al-Y-M and Al-La-M (M = Fe, Co, Ni or Cu) Systems. Jpn. J. Appl. Phys. 27, 280–282 (1988).

[b15] Wang Q., Li J. H., Liu J. B. & Liu B. X. Favored Composition Design and Atomic Structure Characterization for Ternary Al–Cu–Y Metallic Glasses via Proposed Interatomic Potential. J. Phys. Chem. B 118, 4442–4449 (2014). 24735222 10.1021/jp502167t

[b16] Cheng Y. Q. & Ma E. Atomic-level structure and structure–property relationship in metallic glasses. Prog. Mater. Sci. 56, 379–473 (2011).

[b17] Yavari A. R. Materials science: A new order for metallic glasses. Nature 439, 405–406 (2006). 16437101 10.1038/439405a

[b18] Cheng Y. Q., Ma E. & Sheng H. W. Alloying strongly influences the structure, dynamics, and glass forming ability of metallic supercooled liquids. Appl. Phys. Lett. 93, 111913 (2008).

[b19] Wang Q. *et al.* The atomic-scale mechanism for the enhanced glass-forming-ability of a Cu-Zr based bulk metallic glass with minor element additions. Sci. Rep. 4, 4648 (2014). 24721927 10.1038/srep04648 PMC3983660

[b20] Miracle, D. B. A structural model for metallic glasses. Nat. Mater. 3, 697–702 (2004). 15378050 10.1038/nmat1219

[b21] Sheng H. W., Luo W. K., Alamgir F. M., Bai J. M. & Ma E. Atomic packing and short-to-medium-range order in metallic glasses. Nature 439, 419–425 (2006). 16437105 10.1038/nature04421

[b22] Liu X. J. *et al.* Metallic Liquids and Glasses: Atomic Order and Global Packing. Phys. Rev. Lett. 105, 155501 (2010). 21230918 10.1103/PhysRevLett.105.155501

[b23] Ma D., Stoica A. D. & Wang X. L. Power-law scaling and fractal nature of medium-range order in metallic glasses. Nat. Mater. 8, 30–34 (2009). 19060888 10.1038/nmat2340

[b24] Hirata A. *et al.* Direct observation of local atomic order in a metallic glass. Nat. Mater. 10, 28–33 (2011). 21102454 10.1038/nmat2897

[b25] Li J. H., Dai Y. & Dai X. D. Long-range n-body potential and applied to atomistic modeling the formation of ternary metallic glasses. Intermetallics 31, 292–320 (2012).

[b26] Li J. H., Dai X. D., Wang T. L. & Liu B. X. A binomial truncation function proposed for the second-moment approximation of tight-binding potential and application in the ternary Ni–Hf–Ti system. J. Phys.: Condens. Matter 19, 086228 (2007).

[b27] Rosato V., Guillope M. & Legrand B. Thermodynamical and structural properties of f.c.c. transition metals using a simple tight-binding model. Philos. Mag. A 59, 321–336 (1989).

[b28] Cleri F. & Rosato V. Tight-binding potentials for transition metals and alloys. Phys. Rev. B 48, 22–33 (1993). 10.1103/physrevb.48.22 10006745

[b29] Frenkel D. & Smit B. Understanding molecular simulation: from algorithms to applications. (Academic press, 2001).

[b30] Lide D. R. & Bruno T. J. CRC handbook of chemistry and physics. (CRC PressI Llc, 2012).

[b31] Kittel C. & McEuen P. Introduction to solid state physics. Vol. 7 (Wiley, 1996).

[b32] Wang Y. *et al.* Ab initio lattice stability in comparison with CALPHAD lattice stability. Calphad. 28, 79–90 (2004).

[b33] Ellner M., Kattner U. & Predel B. Konstitutionelle und strukturelle untersuchungen im aluminiumreichen teil der systeme Ni-Al und Pt-Al. J. Less Common Metals 87, 305–325 (1982).

[b34] Sridharan S., Nowotny H. & Wayne S. Investigations within the quaternary system titanium-nickel-aluminium-carbon. Monatshefte für Chemie/Chemical Monthly 114, 127–135 (1983).

[b35] Shiga M., Wada H., Nakamura H., Yoshimura K. & Nakamura Y. Characteristic spin fluctuations in Y(Mn_1−x_Al_x_)_2_. J. Phys. F: Metal Phys. 17, 1781 (1987).

[b36] Dagerhamn T. The Crystal Structures of Y_3_Al_2_ and YAl. Acta Chem. Scand. 17, 267 (1963).

[b37] Buschow K. H. J. & Van Der Goot A. S. The crystal structure of rare-earth aluminium compounds R2Al. J. Less Common Metals 24, 117–120 (1971).

[b38] Beaudry B. J., Haefling J. F. & Daane A. H. Some Laves phases of yttrium with transition elements. Acta Crystallogr. 13, 743–744 (1960).

[b39] Lemaire R. & Paccard D. Bulletin de la société française de minéralogie et de cristallographie 90, 311–315 (1967).

[b40] Rose J. H., Smith J. R., Guinea F. & Ferrante J. Universal features of the equation of state of metals. Phys. Rev. B 29, 2963–2969 (1984).

[b41] Liu B. X., Lai W. S. & Zhang Q. Irradiation induced amorphization in metallic multilayers and calculation of glass-forming ability from atomistic potential in the binary metal systems. Mater. Sci. Eng. R. 29, 1–48 (2000).

[b42] Sheng H. W., Wilde G. & Ma E. The competing crystalline and amorphous solid solutions in the Ag–Cu system. Acta Mater. 50, 475–488 (2002).

[b43] Panagiotopoulos A. Z., Quirke N., Stapleton M. & Tildesley D. J. Phase equilibria by simulation in the Gibbs ensemble. Mol. Phys. 63, 527–545 (1988).

[b44] Panagiotopoulos A. Z. Direct determination of phase coexistence properties of fluids by Monte Carlo simulation in a new ensemble. Mol. Phys. 61, 813–826 (1987).

[b45] Li J. H., Zhao S. Z., Dai Y., Cui Y. Y. & Liu B. X. Formation and structure of Al-Zr metallic glasses studied by Monte Carlo simulations. J. Appl. Phys. 109, 113538 (2011).

[b46] Yang B. J. *et al.* Al-rich bulk metallic glasses with plasticity and ultrahigh specific strength. Scripta Mater. 61, 423–426 (2009).

[b47] Inoue A., Matsumoto N. & Matsumoto T. Al-Ni-Y-Co Amorphous Alloys with High Mechanical Strengths, Wide Supercooled Liquid Region and Large Glass-Forming Capacity. Mater. Trans., JIM 31, 493–500 (1990).

[b48] Sanders W. S., Warner J. S. & Miracle D. B. Stability of Al-rich glasses in the Al–La–Ni system. Intermetallics 14, 348–351 (2006).

[b49] Yang B. J., Yao J. H., Chao Y. S., Wang J. Q. & Ma E. Developing aluminum-based bulk metallic glasses. Philos. Mag. 90, 3215–3231 (2010).

[b50] Sun S. P., Yi D. Q., Liu H. Q., Zang B. & Jiang Y. Calculation of glass forming ranges in Al–Ni–RE (Ce, La, Y) ternary alloys and their sub-binaries based on Miedema’s model. J. Alloy. Compd. 506, 377–387 (2010).

[b51] Sheng H. W., Cheng Y. Q., Lee P. L., Shastri S. D. & Ma E. Atomic packing in multicomponent aluminum-based metallic glasses. Acta Mater. 56, 6264–6272 (2008).

[b52] Wang Q., Li J. H., Liu J. B. & Liu B. X. Structural skeleton of preferentially interpenetrated clusters and correlation with shear localization in Mg-Cu-Ni ternary metallic glasses. Phys. Chem. Chem. Phys. 16, 19590–19601 (2014). 25110190 10.1039/c4cp02133a

[b53] Fang X. W. *et al.* Spatially Resolved Distribution Function and the Medium-Range Order in Metallic Liquid and Glass. Sci. Rep. 1, 194 (2011). 22355709 10.1038/srep00194 PMC3245321

[b54] Lee M., Lee C.-M., Lee K.-R., Ma E. & Lee J.-C. Networked interpenetrating connections of icosahedra: Effects on shear transformations in metallic glass. Acta Mater. 59, 159–170 (2011).

[b55] Segall M. D. *et al.* First-principles simulation: ideas, illustrations and the CASTEP code. J. Phys.: Condens. Matter 14, 2717–2744 (2002).

[b56] Clark S. J. *et al.* First principles methods using CASTEP. Z. Kristallogr. -Cryst. Mater. 220, 567–570 (2005).

[b57] Perdew J. P. *et al.* Atoms, molecules, solids, and surfaces: Applications of the generalized gradient approximation for exchange and correlation. Phys. Rev. B 46, 6671–6687 (1992). 10.1103/physrevb.46.6671 10002368

[b58] Perdew J. P., Burke K. & Ernzerhof M. Generalized Gradient Approximation Made Simple. Phys. Rev. Lett. 77, 3865–3868 (1996). 10062328 10.1103/PhysRevLett.77.3865

[b59] Vanderbilt D. Soft self-consistent pseudopotentials in a generalized eigenvalue formalism. Phys. Rev. B 41, 7892–7895 (1990). 10.1103/physrevb.41.7892 9993096

[b60] Monkhorst H. J. & Pack J. D. Special points for Brillouin-zone integrations. Phys. Rev. B 13, 5188–5192 (1976).

